# Interplay between inflammatory bowel disease therapeutics and the gut microbiome reveals opportunities for novel treatment approaches

**DOI:** 10.20517/mrr.2023.41

**Published:** 2023-09-26

**Authors:** Catherine O’Reilly, Susan Mills, Mary C. Rea, Aonghus Lavelle, Subrata Ghosh, Colin Hill, R. Paul Ross

**Affiliations:** ^1^Food Biosciences Department, Teagasc Food Research Centre, Moorepark, Fermoy, Co. Cork P61C996, Ireland.; ^2^Microbiology Department, University College Cork, Co. Cork T12TP07, Ireland.; ^3^APC Microbiome Ireland, University College Cork, Co. Cork T12YT20, Ireland.; ^#^Authors contributed equally.

**Keywords:** Inflammatory bowel disease, gut microbiome, IBD therapeutics, anti-inflammatory therapies, immunosuppressive therapies, responders, non-responders

## Abstract

Inflammatory bowel disease (IBD) is a complex heterogeneous disorder defined by recurring chronic inflammation of the gastrointestinal tract, attributed to a combination of factors including genetic susceptibility, altered immune response, a shift in microbial composition/microbial insults (infection/exposure), and environmental influences. Therapeutics generally used to treat IBD mainly focus on the immune response and include non-specific anti-inflammatory and immunosuppressive therapeutics and targeted therapeutics aimed at specific components of the immune system. Other therapies include exclusive enteral nutrition and emerging stem cell therapies. However, in recent years, scientists have begun to examine the interplay between these therapeutics and the gut microbiome, and we present this information here. Many of these therapeutics are associated with alterations to gut microbiome composition and functionality, often driving it toward a “healthier profile” and preclinical studies have revealed that such alterations can play an important role in therapeutic efficacy. The gut microbiome can also improve or hinder IBD therapeutic efficacy or generate undesirable metabolites. For certain IBD therapeutics, the microbiome composition, particularly before treatment, may serve as a biomarker of therapeutic efficacy. Utilising this information and manipulating the interactions between the gut microbiome and IBD therapeutics may enhance treatment outcomes in the future and bring about new opportunities for personalised, precision medicine.

## INTRODUCTION

Inflammatory bowel disease (IBD) is characterised by recurring chronic inflammation of the gastrointestinal tract (GIT) and is divided into two main disorders, Crohn’s disease (CD) and ulcerative colitis (UC). CD can affect any part of the GIT, though it is usually associated with the large and small intestines, while UC refers to recurring inflammation of the large intestine and rectum^[1]^. Symptoms of CD and UC include abdominal pain, fever, constipation or diarrhoea, and passage of blood and/or mucus, though the passage of blood is more common for UC, while CD is often associated with weight loss^[2,3]^.

Most recent global data suggest that in 2019, 4.9 million people suffered from IBD, an increase of 47.45% between 1990 and 2019^[4]^. In the past, IBD was considered a disease of Western societies; however, now its incidence is growing as more countries become industrialised^[5]^.

While the exact aetiology of IBD is unknown, a combination of genetic susceptibility, environmental factors, an altered immune response, shifts in microbial composition, or loss of tolerance to resident gut microbes is thought to be involved. In terms of gut microbes, compared to healthy individuals, the IBD gut microbiome is characterised by decreased species richness and diversity, a reduction in beneficial bacteria such as bifidobacteria, *Faecalibacterium prausnitzii*, and *Bacteroides* species, and an increase in potentially pathogenic bacteria such as Proteobacteria (e.g., adherent, invasive *Escherichia coli*), *Fusobacterium* species and *Rumincoccus gnavus*, as examples^[6]^. The fungal microbiota also exhibits dysbiosis in IBD. Indeed, disease-specific alterations in diversity indicate that fungi may be favoured at the expense of bacteria in CD^[7]^. IBD subjects have been reported to exhibit an increased gut *Basidiomycota*/*Ascomycota* ratio, increased *Candida albicans* and decreased *Saccharomyces cerevisiae* compared to healthy individuals^[7]^. Faecal concentrations of the gut microbial metabolites, short chain fatty acids (SCFAs), are reduced in active IBD^[8]^. SCFAs are derived from “microbiota-accessible carbohydrates” that include dietary fibre^[9]^ and are the end-products of microbial fermentation. They are involved in several essential cellular and regulatory functions in the human body. For example, SCFA butyrate is the main source of energy for gut epithelial cells^[10]^, and it also has anti-inflammatory properties^[11]^. Propionate activates intestinal gluconeogenesis^[12]^, while acetate has been shown to promote intestinal IgA responses to gut microbiota^[13]^. Thus, changes to the levels of SCFAs produced in the gut can negatively impact physiological processes with deleterious consequences for the host. Bile acid metabolism, another important function of the gut microbiome^[14]^, is dysregulated in IBD, with rates of bacterially-produced secondary bile acids significantly lower in faecal samples from IBD patients while sulphated bile acids are higher^[15]^. Based on *in vitro* experiments, the anti-inflammatory properties of secondary bile acids are abolished upon their sulphation^[15]^. To add to this, secondary bile acids have been associated with anti-inflammatory properties in a mouse model of colitis^[16]^, indicating that bile acid dysmetabolism could impact inflammation in IBD.

There is no single pharmaceutical treatment for UC or CD, given that both are multifaceted disorders. Therapeutics that are used to treat IBD are generally designed to reduce inflammation, thus fostering mucosal healing, and focus on the immune system. These include non-specific anti-inflammatory and immunosuppressive therapeutics such as 5-aminosalicylic acid (5-ASA), methotrexate, glucocorticoids and calcineurin inhibitors, the targeted oral synthetic drug, tofacitinib, and injectable biologics [tumour necrosis factor (TNF) inhibitors, anti-integrin therapy, and anti-interleukin- (IL-)12/23-p40 agents] that target specific components of the immune system. When such conventional treatments fail to work, stem cell therapies, including hematopoietic stem cell transplantation (HSCT) and mesenchymal stem cell therapy (MSCT), have been shown to improve clinical disease. In pediatric CD patients, exclusive enteral nutrition (EEN) is the first treatment choice with proven efficacy.

In recent years, scientists have begun to examine how these therapeutics impact the gut microbiome - an essential consideration given that the gut microbiome is involved in disease pathogenesis. But the impact of the gut microbiome on these therapeutics is also gaining attention, and indeed, research now shows that the gut microbiome itself may improve therapeutic efficacy in some cases but may also hinder efficacy in others. It not only impacts orally delivered therapeutics but also injectable therapeutics since they reach the intestines through enterohepatic circulation. Furthermore, the microbiota could provide a biomarker of treatment efficacy and even recovery for specific therapeutics.

**Figure 1:**
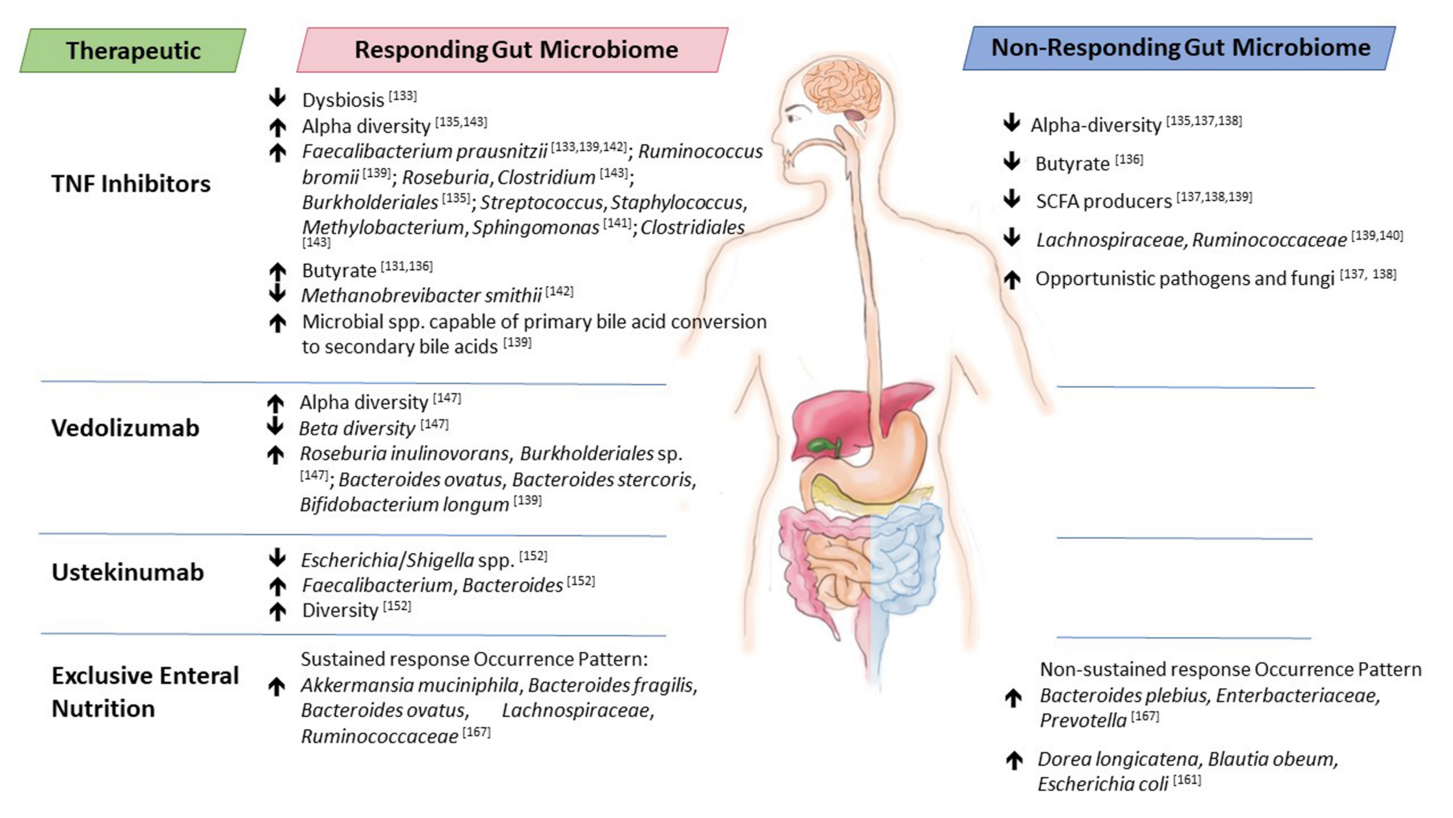
Baseline gut microbiota differences between responders and non-responders to therapeutics used to treat IBD. IBD: Inflammatory bowel disease.

In this review, we provide current knowledge on the interplay between IBD therapeutics and the gut microbiome - how the microbiome can alter IBD therapeutics to positively impact efficacy or contribute to limited performance and the therapeutic-associated changes to gut microbiome composition and functionality [Table 1]. For certain IBD therapeutics, the microbiome composition, particularly before treatment, may serve as a biomarker of efficacy and this is highlighted here [Figure 1], although causality has yet to be identified in these associations. While further studies are warranted with larger cohort sizes to confirm findings, results to date suggest that current IBD therapeutics are impacted by the gut microbiome and vice versa. Thus, future IBD therapies should be developed that focus not only on altering the immune response in IBD but also on gut microbiome composition and functionality to improve therapeutic treatment efficacies in the form of personalised, precision medicine.

**Table 1:** Overview of the interplay between IBD therapeutics and gut microbiome and future possibilities/recommended actions

**Drug**	**Impact of gut microbiota on therapeutic**	**Impact of therapeutic on gut microbiota**	**Future possibilities/recommended actions**
5-ASA	-Bacterial azoreductases release active moiety^[18]^ -Acetylates 5-ASA to ineffective acetyl-5-ASA^[28,29]^	-Inhibits growth of certain species including *Bacteroides* sp., *Clostridium* sp.^[32]^, *Campylobacter concisus*, and *Escherichia coli*^[33]^ -Exacerbates growth of certain *C. concisus* strains^[33]^ -Downregulates *Salmonella* invasiveness^[38]^ -Inhibits bacterial polyphosphate kinase^[39]^ -Increases SCFA-producers, decreases Proteobacteria^[43]^ -Alters Firmicutes: Bacteroidetes ratio^[46]^ -Alters fungal microbiota diversity and composition, restores bacteria-fungi correlation patterns^[48]^	-Increase the abundance of azo-reducing bacteria in the gut using specifically designed “azo-reducing” probiotic strains -Develop inhibitor molecules to microbial acetyltransferases -Investigate the impact of 5-ASA on the gut resistome
Methotrexate	-Metabolise methotrexate to methotrexate-PG^[57]^ -Reduce methotrexate-PG to inactive metabolite DAMPA^[58,59]^	-Inhibits bacterial growth^[52,57,65,67]^ -Selects for antimicrobial resistance genes^[70]^	-Investigate the impact of methotrexate on the gut resistome -Develop methotrexate-specific probiotic strains, e.g., *Bacteroides fragilis*, that ameliorate undesirable changes to the gut epithelium
Glucocorticoids	-Metabolise the drug^[78,79]^ -Convert the drug to androgens^[83]^ -May convert the drug to a metabolite that causes proliferation of prostate cancer cells^[86]^	-Alters microbiome composition to a healthier profile^[87,88,90,91]^	-Investigate drug-metabolising capabilities of the microbiome using metabolomics and metatranscriptomics -Assess the safety of resulting metabolites -Identify microbial enzymes responsible for drug metabolism
Calcineurin inhibitors	-Metabolise drug to less potent metabolites^[100]^	-Alters microbiome composition and functionality^[88,101-103]^	-Investigate drug-metabolising capabilities of the microbiome using metabolomics and metatranscriptomics -Assess the safety of resulting metabolites -Identify microbial enzymes responsible for drug metabolism -Develop strategies to prevent drug metabolism
Tofacitinib	-	-Alters microbiome composition^[113]^	-Investigate the impact of gut microbiota on the drug
TNF inhibitors	-May bind to the drug^[122]^ -May cleave the drug^[123]^	-Alters microbiome composition and functionality to a healthier profile^[127,128,130]^	-Investigate the impact of gut microbiota on drug stability -Develop strategies to prevent drug cleavage
Vedolizumab ustekinumab	-	-	-Investigate the impact of gut microbiota on the drugs -Investigate the impact of the drugs on gut microbiota
Exclusive enteral nutrition	-	-Alters microbiome composition^[160,163,166]^ -Can reduce microbial diversity^[157,158,161,162]^ -Improves/normalises bile acid metabolism^[161,163,165]^ -Alters SCFA levels^[166]^	-Administer *Akkermansia muciniphila* before treatment to improve remission maintenance
Hematopoietic stem cell transplantation	-	-Alters microbiome composition to a healthier profile^[173,174]^	-Investigate the impact of gut microbiota on therapy efficacy
Mesenchymal stem cell therapy	-	-Alters microbiome composition to a healthier profile^[187-189]^ -Decreases sulphur metabolism^[189]^ -Increases *Cetobacterium* relative abundance^[190]^	-Investigate the impact of gut microbiota on therapy efficacy

DAMPA: 4-amino-4-deoxy-N-methylpteroic acid; IBD: inflammatory bowel disease; methotrexate-PG: methotrexate-polyglutamate; SCFA: short chain fatty acid; TNF: tumour necrosis factor; 5-ASA: 5-aminosalicylic acid.

## NON-SPECIFIC ANTI-INFLAMMATORY AND IMMUNOSUPPRESSIVE THERAPEUTICS

### 5-aminosalicylic acid

5-ASA, including mesalamine or mesalazine, is used to treat mild to moderate UC^[17]^ and is generally administered orally. It was initially developed in the 1940s as a prodrug linked to sulfapyridine via an azo bond, known as sulfasalazine, where sulfapyridine prevents the absorption of 5-ASA in the small intestine^[18]^. Although developed to treat rheumatoid arthritis, sulfasalazine was soon recognised to be an effective treatment for IBD in patients with mild to moderate disease^[18]^. However, due to the adverse events associated with the sulfapyridine moiety of sulfasalazine, including nausea, headaches, dyspepsia and anorexia^[18,19]^, other 5-ASA products have since been developed that do not require sulfapyridine. Balsalazide and olsalazine are azo-bonded 5-ASA prodrugs, but the former is composed of 5-ASA bonded with 4-aminobenzoyl-β-alanine, while the latter consists of two azo-bonded 5-ASA molecules^[20]^.

According to the Biopharmaceutics Classification System (BCS), 5-ASA itself is categorized as a BCS class IV drug (low solubility, low permeability). It is slightly soluble in water and, following its oral administration, is poorly absorbed (approximately 25%)^[21]^. Most of the oral 5-ASA products on the market have a pH-dependent coat, which enables them to withstand the release of 5-ASA in the stomach (which has a low pH) and be released in the intestine with a higher pH^[20,22]^.

The mechanism of action of 5-ASA for the treatment of IBD is still not completely understood; however, it is thought to downregulate inflammation and reduce inflammatory cytokine release by activating nuclear receptors (i.e., the gamma form of peroxisome proliferator‐activated receptors (PPARs)^[23]^. It affects a range of signalling pathways and mediators which are involved in leukocyte chemotaxis and function (eicosanoids, IL-8, NF-kB, and PPAR-γ) and epithelial defence (scavenging free radicals, fortifying epithelial resistance to injury, promoting cellular proliferation and inhibition of apoptosis)^[24]^.

### Impact of the gut microbiota on 5-ASA

Azo-bonded therapeutics depend on the gut bacteria for activation, as they are split in the colon by bacterial azoreductases to release the active 5-ASA moiety^[18]^. Azoreduction is considered to be a ubiquitous function within the gut microbiome, and a recent study reported potentially uncharacterised gut microbiota azoreductases based on a search of the 4,644 genome sequences in the representative Unified Human Gastrointestinal Genomes collection^[25]^. However, another study reported that the total relative abundances of azo-reducing bacteria for the majority of individuals (healthy and IBD patients) fell below 3% such that the authors concluded that gut microbiota azoreductases are sparse in both health and disease, a finding that was further supported by the lack of azoreductases in the complementary metatranscriptomics and metaproteomics datasets^[26]^. The authors conclude that poor efficacies and idiopathic responses associated with azo-linked prodrugs could be partially attributed to the low levels of gut microbial azoreductases^[26]^. Yet, using an *in vitro* colonic simulator, Sousa *et al*. reported that individual faecal slurries were capable of degrading sulfasalazine within 4 h^[27]^. In the same study, pooled faecal slurries degraded sulfasalazine much faster (2 h) than other azo-bonded prodrugs of 5-ASA, including balsalazide (3 h) and olsalazine (> 4 h).

However, some gut bacteria can acetylate 5-ASA^[28,29]^, as can the intestinal mucosa^[30]^, and the resulting acetyl 5-ASA is ineffective for treating IBD^[28]^. Until recently, the microbial enzymes responsible remained unknown. Indeed, a multi-omics work-flow consisting of gut microbiome metagenomics, metabolomics and metatranscriptomics identified 12 previously uncharacterised microbial acetyltransferases capable of acetylating 5-ASA which belong to two protein superfamilies, namely thiolases and acyl-CoA N-acetyltransferases^[31]^. The presence of three thiolases and one acyl-CoA N-acetyltransferase in the human gut microbiome was associated with an increased risk of treatment failure of 5-ASA based on the analysis of the discovery cohort and the SPARC IBD cohort (Study of a Prospective Adult Research Cohort with IBD)^[31]^. While this relationship needs to be confirmed with larger cohorts of patients, it does pave the way toward future “personalised microbiome-based medicine”. Indeed, the authors suggest that 5-ASA-modifying thiolase sequences in the gut microbiome of a patient could be used as a biomarker of treatment efficacy and that microbiome-specific inhibitors of enzymes such as thiolases could be developed to enhance 5-ASA treatment efficacy^[31]^.

### Impact of 5-ASA on the gut microbiota

A number of studies have investigated the impact of 5-ASA on the gut microbiota. *Ex vivo* studies have found that it can inhibit the growth of gut *Bacteroides* and *Clostridium* species grown in pure culture^[32]^. 5-ASA has also been shown to inhibit some strains of *Campylobacter concisus* while promoting the growth of other *C. concisus* strains and it can inhibit *E. coli*^[33]^. Interestingly, *C. concisus* is believed to initiate a subgroup of IBD^[34,35]^. Moreover, 5-ASA has, in the past, been reported to exacerbate some cases of IBD^[36]^. Thus, Liu *et al*. propose that 5-ASA-related worsening of IBD symptoms could be due to 5-ASA promoting the growth of certain IBD-causing bacteria in the gut^[33]^. 5-ASA has been shown to inhibit IBD- and colorectal cancer-associated *E. coli* in a dose-dependent manner and it down-regulated *E. coli* virulence genes associated with both diseases^[37]^. *E. coli* motility was inhibited by 30%-70% in the presence of 5-ASA and it also inhibited *E. coli* adherence to and invasion of Caco-2 cells while promoting a cellular anti-inflammatory response. Kaufman *et al*. studied the effect of 5-ASA on bacterial gene expression of *Salmonella enterica* serovar Typhimurium^[38]^. They found that 5-ASA downregulated invasiveness genes in *Salmonella*, which correlated with the ability of 5-ASA to attenuate the invasiveness of *S. enterica* serovar Typhimurium exposed to cultured epithelial monolayers.

Interestingly, 5-ASA has been shown to decrease polyphosphate levels in a diverse range of bacteria, including gut bacteria, by inhibiting bacterial polyphosphate kinase^[39]^. Polyphosphates are made up of multiple phosphate monomers and are involved in virulence, persistence, biofilm formation, and oxidative stress^[40,41]^, and have been shown to help bacteria evade host immunity by interfering with macrophages^[42]^. 5-ASA inhibition of bacterial polyphosphate kinase has been shown to reduce bacterial colonisation and biofilm formation and sensitize bacteria to oxidative stress and has been suggested as another mode of action in the treatment of IBD^[39]^.

Zheng *et al*. investigated the impact of sulfasalazine on the gut microbiota in experimental colitis in rats induced by 2,4,6-trinitrobenzenesulfonic acid (TNBS)^[43]^. Sulfasalazine restored the TNBS-induced gut microbiota dysbiosis as evidenced by the increase in SCFA-producing bacteria and decrease in Proteobacteria, and it also restored gut microbiome functionality, decreased oxidative stress and bacterial pathogenesis. 5-ASA ameliorated dextran sulfate sodium (DSS)-induced colitis in mice by activating PPAR-γ signalling in the colonic epithelium^[44]^. Interestingly, the DSS-associated reduction of mitochondrial activity in the intestinal epithelium was associated with increased oxygen which fuelled the expansion of *E. coli*. However, the activity of 5-ASA restored mitochondrial activity through PPAR-γ signalling and prevented *E. coli* expansion. In DSS-induced colitis in piglets, 5-ASA alleviated symptoms, decreased diamine oxidase activity and D-lactate levels, improved mucosal damage, reduced the colon macrophage CD11b^+^ and CD3^+^ T-cell infiltrations, and was also found to decrease the abundance of methanogens^[45]^.

Andrews *et al*. measured the effect of 5-ASA treatment on faecal microbiota composition, mucosal proteolytic activity, and clinical efficacy in 14 patients with irritable bowel syndrome-diarrhoea (IBS-D)^[46]^. 5-ASA treatment caused the responding patients to have a more similar faecal microbiota to each other than non-responders. Before treatment, 95% of the microbiota in the patients’ stool samples were made up of Firmicutes and Bacteroidetes. Following 5-ASA treatment, there was an increase in the abundance of Firmicutes and a decrease in Bacteroidetes. These changes were not statistically significant, however (possibly due to the small number of patients in the study). The clinical benefits observed were not sustained after the four-week treatment period and the changes in gut microbiota composition returned to levels comparable to pretreatment levels. Patients with quiescent UC in receipt of 5-ASA therapies and showing high levels of mucosal 5-ASA had a reduced abundance of mucosal pathogenic bacteria such as Proteobacteria and an increased abundance of health-associated bacteria such as *Faecalibacterium*; this correlation was not observed for faecal bacteria^[47]^. 5-ASA has also been shown to alter fungal microbiota diversity and composition in UC patients, and it was able to restore bacteria-fungi correlation patterns^[48]^.

## METHOTREXATE

Methotrexate is an immunosuppressive drug that has been used effectively for over 20 years in patients with CD. It is recommended for use in combination with anti-TNF agents^[49]^. It is an analogue of dihydrofolate; thus, it inhibits dihydrofolate reductase - preventing the synthesis of purines and pyrimidines, the building blocks of DNA synthesis. Methotrexate can be administered orally or parenterally, where the latter is the preferred option, as injection is deemed more effective with fewer side effects^[50]^. Parenterally-administered methotrexate can also interact with the intestinal microbiota due to biliary secretion^[51,52]^. Within the cells, methotrexate undergoes polyglutamation through the addition of varying numbers of glutamic acid molecules, resulting in a much more active version of the drug, methotrexate-polyglutamate (methotrexate-PG), where the longer-chain methotrexate-PGs are more potent^[53,54]^. Because of its ability to inhibit nucleic acid synthesis in rapidly proliferating cells, it was initially used in oncology^[53]^. However, it is used at lower concentrations to treat IBD, where it exerts an anti-inflammatory response through inhibition of the enzyme 5-aminoimidazole-4-carboxamide ribonucleotide formyltransferase/inosine monophosphate cyclohydrolase (ATIC)^[55]^. Inhibition of ATIC results in the excretion of accumulated adenosine from the cell, which then binds to G-protein coupled receptors on target cells, inducing an anti-inflammatory cascade^[56]^.

### Impact of gut microbiota on methotrexate

In 2016, a conference abstract reported that out of 25 gut bacterial isolates, two were capable of metabolizing methotrexate into methotrexate-PG^[57]^. However, Sayers *et al*. state that given that methotrexate-PG is not as easily transported as methotrexate from cells, intestinal bacterial cells will more than likely retain methotrexate-PG or reconvert it to methotrexate before export; thus, bacterially-generated methotrexate-PG is unlikely to influence methotrexate treatment efficacy^[55]^.

The efficacy of methotrexate-PG can be reduced through the removal of glutamate entities via carboxypeptidases^[55]^, resulting in the inactive metabolite 4-amino-4-deoxy-N-methylpteroic acid (DAMPA)^[58,59]^. Such an enzyme was isolated from a strain of *Pseudomonas stutzeri*, named carboxypeptidase G2 (CPG2)^[58,60]^. The enzyme has been developed to prevent methotrexate-PG toxicity in patients due to high doses as it rapidly decreases methotrexate-PG levels in the blood^[55,61]^. Other bacterial species have been shown to harbour orthologs of CPG2 including *E. coli* (*p*-aminobenzoyl-glutamate hydrolase)^[62]^. Shin *et al*. showed that *Lactobacillus casei* (now *Lacticaseibacillus casei*) was also able to metabolize methotrexate mainly to DAMPA^[63]^. However, Sayers *et al*. state that bacterial removal of glutamate from methotrexate-PG would be unlikely to decrease the efficacy of treatment as it would not impact already-absorbed and modified methotrexate-PG in eukaryotic cells, particularly for parenterally-administered methotrexate^[55]^.

### Impact of methotrexate on gut microbiota

Methotrexate is structurally similar to the antibiotic trimethoprim, which is also an analogue of dihydrofolate, although trimethoprim predominantly targets prokaryotic dihydrofolate reductases^[64]^. However, methotrexate has been reported to inhibit microbial growth. In combination with 6-mercaptopurine, methotrexate was shown to inhibit the growth of *Mycobacterium avium* subspecies *paratuberculosis in vitro*^[65]^, which is believed to be a causative agent of idiopathic IBD^[66]^. Methotrexate was reported to inhibit the growth of 19 out of 25 gut isolates^[57]^. The same research group later reported that although drug sensitivity varied across strains, the mechanism of action against bacterial cells was similar to that against mammalian cells - the inhibition of dihydrofolate reductase^[52]^. In the same study, methotrexate was found to decrease Bacteroidetes in gnotobiotic mice colonised with human gut microbiota. Furthermore, *in vitro* methotrexate treatment of faecal samples from rheumatoid arthritis patients divided into responders and non-responders to methotrexate revealed that while both microbiotas were sensitive to methotrexate, the changes in bacterial taxa and gene family abundance were distinctly different between the two groups. The authors propose that the changes to the gut microbiome could have subsequent consequences for host immunity^[52]^. Indeed, transplantation of methotrexate-treated stool samples from responding patients into inflammatory-triggered germ-free mice resulted in reduced immune activation.

Other studies have reported the impact of methotrexate on the Bacteroidetes phylum^[59,67]^. Interestingly, a significant decrease in *Bacteroides fragilis* was observed in mice treated with methotrexate and consequently suffering mucosal injury which appeared to be inversely proportionate to macrophage density^[67]^. However, the gavage of the mice with *B. fragilis* ameliorated the inflammatory damage induced by methotrexate and normalised macrophage levels. This finding may be of significance to patients who experience methotrexate-induced colitis even at low doses. While this is rare, a recent case report described the development of colitis and pancytopenia in a patient receiving chronic low-dose methotrexate^[68]^.

A number of laboratory strains of *E. coli* have been found to be resistant to methotrexate due to the expression of a TolC-dependent AcrAB multidrug resistance efflux pump^[69]^. Furthermore, exposure of *E. coli* and *Klebsiella pneumoniae* strains to methotrexate selected for acquired trimethoprim resistance genes on a chromosome or plasmid^[70]^. It also co-selected for genetically linked resistance genes when co-present with trimethoprim resistance on a multidrug resistance plasmid. These effects occurred at low concentrations, 40- to > 320-fold below the minimum inhibitory concentration of methotrexate. This is a worrying concern for patients receiving this drug since its use could cause expansion/amplification of the gut microbial resistome and could impact the efficacy of future antimicrobial therapies in patients.

## GLUCOCORTICOIDS

Glucocorticoids are a class of corticosteroid hormones. They are generated in the human body in the adrenal cortex and secreted into the blood. They are involved in several essential physiological functions, from regulation of immunity to metabolism of fat, glucose, and protein, as examples^[71,72]^. They can also be synthesised in the laboratory. Prednisone (prodrug of prednisolone) and methylprednisolone are first-generation glucocorticoids used to treat IBD, while budesonide and beclomethasone dipropionate represent second-generation glucocorticoids for IBD treatment^[73,74]^. The second-generation drugs have less severe adverse effects than the first-generation glucocorticoids^[73,74]^.

Glucocorticoids are effective in inducing remission in IBD^[75]^ and can be administered orally, parenterally, or rectally. In the current European Crohn’s and Colitis Organization (ECCO) guidelines, systemic corticosteroids are recommended in patients with active, moderate-to-severe CD and UC for the induction of clinical response and remission^[76]^. However, glucocorticoids are not effective for the maintenance of remission, and long-term use should be avoided due to the risk of adverse events or comorbidities^[77]^.

Glucocorticoids work by binding to the glucocorticoid receptor in the cytoplasm of immune cells^[74]^. This results in a conformational change of the glucocorticoid receptor and the translocation of the complex to the cell nucleus, where it regulates the expression of genes involved in inflammation, resulting in an anti-inflammatory cascade.

### Impact of gut microbiota on glucocorticoids

Incubation of prednisone with a human faecal inoculum resulted in 9 different metabolites^[78]^. Computer-aided prediction revealed that the majority of the metabolites could be capable of inhibiting prostaglandin E2 9-ketoreductase, an enzyme found in a variety of animal species and tissues, although further studies are warranted to confirm this. Yadav *et al*. investigated the impact of a mixed human faecal inoculum in simulated colonic conditions on a number of glucocorticoid drugs including prednisolone, budesonide, beclomethasone (17,21) dipropionate and its active metabolite beclomethasone (17) monopropionate, each at three different concentrations, 0.00274, 0.0137, and 0.0274 mM, which represent the typical oral drug doses^[79]^. Within 3 h, prednisolone was completely degraded at all concentrations; likewise, beclomethasone (17,21) dipropionate was degraded within 2 h, while its active metabolite remained stable. Budesonide was degraded within 7 h. In the absence of the faecal inoculum, all drugs were stable. Another study reported that the degradation of budesonide in the distal small intestine and proximal colon, based on *in vitro* analysis by incubating with different dilutions of faecal material, would be clinically irrelevant since the degradation half-life was estimated to be 203 and 147 min, respectively^[80]^, while the estimated fraction absorbed after colonic administration was shown to be 101% in humans^[81]^.

A few studies have investigated the bacterial enzymes involved in glucocorticoid degradation. The gut microbe *Clostridium scindens* has been found to be capable of degrading prednisone^[82]^ and converting glucocorticoids to androgens by cleaving the side chain^[83]^. A cortisol-inducible operon was located in *C. scindens* that encoded at least one enzyme involved in side-chain cleavage, including steroid-17,20-desmolase, although phylogenetic analysis suggested that the encoding-operon is rare^[83]^. Steroid-17,20-desmolase only works under anaerobic conditions such as those in the gut^[84,85]^. Ly *et al*. also reported that bacterial steroid-17,20 desmolase is taxonomically rare but proved that it converts prednisone to 1,4-androstanediene-3,11,17-trione, a metabolite that causes the proliferation of prostate cancer cells^[86]^. This finding warrants further investigation into the impact of the gut microbiome on prednisone using a combination of approaches including shotgun metagenomics, metatranscriptomics, and metabolomics and an assessment of the safety of the resulting metabolites.

### Impact of glucocorticoids on gut microbiota

A study in adult male C57B1/6 mice reported that injection with synthetic glucocorticoid, dexamethasone (DEX), once daily for 10 days (representing acute treatment) or once every 3 days over a four-week period (chronic treatment) resulted in substantial shifts in the gut microbiota for both treatments^[87]^. Specifically, the genus *Bifidobacterium* was significantly elevated after DEX treatment and *Lactobacillus* was also increased, while the colonic mucin degrader, *Mucispirillum*, was absent after DEX treatment. Transferring microbiota from DEX-treated mice to a genetically susceptible mouse model of colonic inflammatory disorders (IL-10 knockout mice) ameliorated inflammation, suggesting that the DEX-induced microbiota changes are a factor in its efficacy. Another study in mice revealed that 14 days of prednisolone treatment resulted in disruption to the faecal microbiota community by decreasing Bacteroidetes and increasing Firmicutes^[88]^. Prednisolone was associated with dramatic reductions in ileal levels of *Clostridium sensu stricto* which correlated with altered ileal expression of C-type lectins and IL-22. Prednisolone also resulted in alterations to the faecal microbiota at the family level, though changes were non-consistent between replicates. A combination of prednisolone with two other immunosuppressive drugs, mycophenolate mofetil and tacrolimus, was also associated with reduced *Clostridium sensu stricto* in the ileum. The combined treatment also enabled a commensal *E. coli* to flourish and increased colonization of a uropathogenic strain of *E. coli*.

In healthy dogs, prednisolone administration for 14 days had no impact on faecal bacterial composition or diversity based on 16s rRNA metagenomic sequencing^[89]^. In contrast, in dogs with IBD, prednisone was associated with significant alterations in the spatial distribution of mucosal bacteria, resulting in elevated levels of bifidobacteria and streptococci across all mucosal apartments, while increasing bifidobacteria, faecalibacteria, and streptococci in adherent mucus^[90]^. Furthermore, the drug increased the expression of the tight junction proteins occludin and E-cadherin while reducing zonulin.

In humans with microscopic colitis, treatment with budesonide drove the composition of the faecal microbiota toward that of healthy individuals during and after treatment^[91]^. More studies are warranted that examine the impacts of these drugs on the gut microbiota.

### Calcineurin inhibitors

Calcineurin inhibitors such as cyclosporine A and tacrolimus are immunosuppressants that work by inhibiting calcineurin, a eukaryotic Ca^2+^- and calmodulin-dependent serine/threonine protein phosphatase^[92]^ and in doing so prevent calcineurin from dephosphorylating the nuclear factor of activated T-cells (NFAT), thus inhibiting T cell activation and blocking the transcription of proinflammatory cytokines^[93]^. Cyclosporine A is a poorly soluble cyclic peptide, while tacrolimus is a macrolide. The efficacy of intravenous cyclosporine treatment in patients with severe UC has been confirmed by several clinical trials^[94-97]^. Tacrolimus has been used in more recent times for the treatment of IBD and has demonstrated “remarkable” efficacy in the treatment of steroid-refractory UC^[98]^. Both tacrolimus and cyclosporine can be administered orally or intravenously.

### Impact of gut microbiota on calcineurin inhibitors

Only a limited number of studies have investigated the impact of gut bacteria on calcineurin inhibitors. However, the results to date reveal that further studies are warranted, particularly in the case of tacrolimus.

In a study investigating the stability of peptide molecules in a model of the large intestine using a mixed human faecal inoculum, cyclosporine proved to be highly stable, with 87% still intact after 120 min exposure^[99]^. Indeed, the degradation rate was directly correlated with peptide lipophilicity. In contrast, tacrolimus was found to be metabolized by commensal gut bacteria, including *F. prausnitzii* and members of *Clostridiales*^[100]^. One of the degradation compounds was identified as a C-9 keto-reduction product of the drug and was 15-fold less potent in terms of immunosuppressive activities than intact tacrolimus. The authors concluded that the gut microbial metabolism of the drug could be contributing to its low oral bioavailability.

### Impact of calcineurin inhibitors on the gut microbiota

In mice, tacrolimus administration was associated with altered faecal microbiota at the family level, although the changes differed between replicates^[88]^. Another study in mice reported that high-dose tacrolimus treatment (10 mg/kg body weight/day by oral gavage - closely resembling serum concentration of the drug in humans) was associated with a significant increase in the relative abundance of 3 taxa, namely *Allobaculum*, *Bacteroides* and *Lactobacillus*^[101]^. It also significantly increased colonic mucosal and circulatory levels of CD4^+^CD25^hi^FoxP3^+^ regulatory T cells. Faecal microbiota transfer from the high-dose tacrolimus donors induced the same immunomodulatory effects in the recipients. Bioinformatic analysis indicated that tacrolimus resulted in altered microbiome functionality with one pathway involved directly or indirectly in the oxidation of the SCFAs, acetate and pyruvate, respectively. The authors propose that the altered microbiota may lead to altered levels of SCFAs, which in turn enhance the anti-inflammatory effects of T-regs, suggesting that the tacrolimus-induced gut microbiota alterations are important in the immunosuppressive effects of the drug. In a more recent study, the same researchers investigated the impact of tacrolimus in a mouse model of DSS-induced colitis^[102]^. The drug (10 mg/kg) significantly ameliorated colitis in mice and induced a “remarkable” expansion of *Lactobacillus*. Furthermore, the administration of *Lactiplantibacillus plantarum* with tacrolimus improved gut microbiome diversity and promoted the therapeutic effect of the drug.

Following liver transplantation in rats, tacrolimus at 0.5 mg/kg body weight was identified as the optimal dose of the drug as it induced immunosuppression, normal graft function, and a stable gut microbiota characterised by increased species richness, reduced *Bacteroides-Prevotella* group and *Enterobacteriaceae*, and increased commensals, *F. prausnitzii* and *Bifidobacterium* species^[103]^. Interestingly, lower and higher concentrations (0.1 and 1.0 mg/kg body weight, respectively) proved suboptimal for maintaining immunosuppression and inducing normal graft function and both concentrations of tacrolimus were associated with decreased species richness, while the low concentration (0.1 mg/kg body weight) was associated with an increase in the *Bacteroides-Prevotella* group and *Enterobacteriaceae* and a decrease in *F. prausnitzii* and *Bifidobacterium* species. Another study in mice also reported that tacrolimus at 1 mg/kg/day i.p. resulted in decreased microbial diversity, increased Firmicutes: Bacteroidetes ratio and decreased acetate and butyrate-producing bacteria^[104]^, highlighting the importance of optimising the dose.

Much research in humans on the impact of calcineurin inhibitors on the gut microbiome is in relation to organ transplantation. Indeed, tacrolimus is commonly administered to transplant patients to prevent rejection of a transplanted organ. The concentration of tacrolimus administered to transplant patients and IBD patients is generally within a similar trough range (10-15 ng/mL blood)^[105,106]^. The impact of tacrolimus on the faecal microbiome was assessed in renal transplant patients in conjunction with the immunosuppressant mycophenolate mofetil^[107]^. Based on a bioinformatics approach, the tacrolimus + mycophenolate mofetil treatment was associated with the enrichment of a flagellar motion switch protein and a type IV pilus assembly protein in faecal microbiome data. The authors suggest that these effects may also have resulted from indirect effects; thus, larger multicentre studies with large sample sizes and healthy controls are required to further investigate the impacts of immunosuppressive drugs on the gut microbiome in humans. Interestingly, encapsulated cyclosporine in a colon-targeted delivery system designed to deliver cyclosporine in a solubilised form had negligible impact on gut microbiota composition in healthy individuals or when assessed in an *ex vivo* colon model^[108]^.

## TARGETED ORAL SYNTHETIC DRUG

### Janus kinase inhibitor (tofacitinib)

Janus kinase (JAK) inhibitors are small synthetic molecules that act intracellularly and can modulate the response of a variety of proinflammatory cytokines implicated in IBD pathogenesis^[109,110]^. Tofacitinib is a JAK inhibitor that has high selectivity for JAK-1 and JAK-3 and has been approved for use by the Food and Drug Administration (FDA) and the European Medicines Agency (EMA).

### Impact of tofacitinib on the gut microbiome

To date, no studies have investigated the impact of the gut microbiome on tofacitinib and very few studies have investigated the impact of tofacitinib on the gut microbiome. In a mouse study investigating the active components of a traditional Chinese medicine on UC, tofacitinib was used as a positive control^[111]^. The authors report that tofacitinib did not result in any obvious changes to the DSS-altered murine gut microbiota following 14 days of treatment. Likewise, short-term treatment with the drug (3.5 days) had minimal impact on the colonic microbiota in a mouse model of colitis^[112]^. In contrast, Hablot *et al*. reported that although tofacitinib was not associated with altered alpha diversity of the gut microbiota in a mouse model of rheumatoid arthritis from day 21 of treatment, it was associated with altered beta diversity^[113]^. In the treated mice, a reduction in potential pathogenic members of the phyla Proteobacteria and Chlamydiae was noted, along with an increase in some beneficial members of Actinobacteria and Firmicutes. The question remains if some of the anti-arthritic effects of the drug are microbiota-driven. Studies are clearly needed to explore the impact of this drug on the gut microbiome in IBD.

## INJECTABLE BIOLOGICAL DRUGS, MONOCLONAL ANTIBODIES

### TNF inhibitors

TNF is a cell signalling cytokine involved in inflammation whose primary role is the regulation of immune cells. TNF signalling drives the expression of acute-phase proteins and influences cell migration, proliferation, and cell death^[114]^. However, TNF can become harmful if over- or inappropriately expressed, resulting in chronic inflammation, eventually leading to autoimmune diseases, and is a prominent pathological cytokine in IBD^[114-117]^. Therapies that target TNF in IBD, namely TNF inhibitors, have been shown to induce mucosal healing, minimize the use of steroids, reduce hospitalizations and surgeries, and improve the overall quality of life in IBD patients^[118]^. TNF inhibitors are immunoglobulin G (IgG) 1 monoclonal antibodies or fragment antigen binding (Fab) antibodies and include infliximab, adalimumab, certolizumab-pegol, and golimumab^[117]^. More specifically, golimumab and adalimumab are human IgG1 antibodies, infliximab is a chimeric IgG1 antibody, while certolizumab-pegol is a PEGylated Fab fragment of a humanized anti-TNF antibody^[119]^. These drugs are intravenously or subcutaneously administered^[120]^. However, the effectiveness of these therapies is limited. Up to 30% of IBD patients are non-responsive to treatment (show no clinical benefit after induction therapy), while a further 30%-40% of patients may lose responsiveness within one year, requiring them to switch to another biologic or require dose-escalation^[121]^. This has been attributed to several factors, from the pharmacokinetics and immunogenicity of the drug itself to patient genetics and disease activity, the location and severity of disease, and/or the treatment strategy (dosage, combination therapy)^[121]^. However, the gut microbiota may also be a contributing factor to the limited effectiveness of this drug.

### Impact of gut bacteria on TNF inhibitors and treatment efficacy

A number of bacterial species have been shown to harbour IgG-binding proteins which have been suggested as additional virulence factors that reduce IgG reactions with the microbial cell^[122]^. In addition, certain bacterial species including *Streptococcus pyogenes*, *Pseudomonas aeruginosa*, *Staphylococcus aureus*, *Proteus mirabilis* and *Treponema denticola* have been shown to harbour enzymes that can cleave IgG1^[123]^. This suggests that members of the gut microbiome could be capable of interfering with the efficacy of TNF inhibitors, especially intramucosal bacteria which are more frequent and more extensively distributed in IBD patients^[124]^. Deveuve *et al*. reported that *S. pyogenes* protease, IdeS, is capable of cleaving monoclonal antibody therapies including infliximab^[125]^. It has been suggested that proteolytic degradation may be responsible for the non-responsiveness of patients, given that high levels of cleaved IgG have been detected in the sera of IBD patients, although this cleavage was caused by matrix metalloproteinases 3 and 12^[126]^. Thus, studies are warranted to determine if microbial cleavage of or binding to TNF inhibitors contributes to treatment non-responsiveness in some patients.

### Impact of TNF inhibitors on the gut microbiota

In paediatric CD patients, infliximab treatment was associated with increased faecal microbiota diversity and shifted its composition and functional capabilities toward a healthy profile^[127]^. The faecal microbiota before treatment showed a significant loss of SCFA-producing bacteria; however, infliximab treatment failed to significantly expand SCFA-producing taxa. Despite this, sustained response to the treatment correlated with the abundance of SCFA producers in the faecal microbiota. In adults, infliximab treatment was also associated with increased faecal microbiota diversity and richness, but, unlike the previous study, it resulted in a significant increase in SCFA-producers while pathogenic bacteria decreased^[128]^. Interestingly, significant increases in *Lachnospiraceae* and *Blautia* during treatment were associated with infliximab efficacy. Ditto *et al*. also reported that treatment of enteropathic arthritis patients with TNF inhibitors was associated with a remarkable increase in faecal levels of *Lachnospiraceae* along with the *Coprococcus* genus while an increased trend in Clostridia was observed, but it was also was associated with a decreasing trend in Proteobacteria and Gammaproteobacteria^[129]^. Successful adalimumab therapy was associated with a significant decrease in Proteobacteria in CD patients, a change that was not observed in non-responding patients^[130]^. Effenberger *et al*. also reported a significant relative decrease in Proteobacteria during treatment with TNF inhibitors in CD patients, along with an increase in Bacteroidetes^[131]^. During 8 weeks of infliximab maintenance therapy in IBD patients in clinical remission, the treatment did not significantly impact faecal microbiota composition, species diversity, or richness between weeks 1 and 7, but at week 7, patients with trough levels of infliximab ≥ 5 μg/mL showed increased species richness while those with mucosal healing showed increased species diversity^[132]^. At the same time period, the relative abundances of certain bacteria were also seen to significantly differ between patients with trough levels of ≥ 5 μg/mL and < 5 µg/mL infliximab, with bacteria such as *F. prausnitzii* and *Bacteroides uniformis* associated with ≥ 5 μg/mL and the *Enterococcus faecium* group associated with < 5 µg/mL.

### Gut microbiome biomarkers of responders and non-responders to TNF inhibitors

Interestingly, scientists have investigated the gut microbiota of responders and non-responders to TNF inhibitors by studying the microbiota before and after treatment to determine the signatures of each. For example, gut microbiota differences were established in UC patients (*n* = 56) at baseline (before commencement of treatment) that differentiated responders from non-responders who underwent anti-TNF therapy^[133]^. Specifically, responders to the infliximab/adalimumab therapy had lower dysbiosis indexes and higher *F. prausnitzii* abundance compared with non-responders, while the relative abundance of *F. prausnitzii* increased in responders during therapy. The American Gastroenterology Association published an abstract that reported that panels of specific gut taxa in UC patients at baseline differentiated responders from non-responders to the TNF inhibitor golimumab at week 6 (*n* = 801) of treatment^[134]^. These panels included *B. fragilis*, *Bacteroides ovatus*, and several members of the *Enterobacteriaceae* family. Furthermore, at the end of treatment, the microbiota of responders was significantly more diverse than non-responders. In patients suffering from spondyloarthritis (*n* = 19), a higher proportion of *Burkholderiales* at baseline was indicative of clinical response to anti-TNF treatments after 3 months of therapy^[135]^. Additionally, the alpha diversity of the gut microbiota of non-responders at baseline was lower than that of responders and partial responders. In contrast, microbiota diversity indices across time points (baseline to week 30 of treatment) did not differ greatly between responding and non-responding IBD patients receiving TNF inhibitors^[136]^. However, in this study, *in silico* modelling to identify metabolic interactions between gut microbes revealed that intercellular exchange of butyrate was reduced by 81% in baseline samples of non-responders compared to responders. In a later study*, in silico* simulations predicted a 1.7-fold greater butyrate production capacity in patients who achieved remission compared to non-responders treated with either TNF inhibitors or azathioprine^[131]^. A lack of SCFA-producing bacteria, increased opportunistic pathogens and fungi and decreased biodiversity have also been identified as microbiome features of non-responding IBD patients to anti-TNF agents^[137,138]^. Along with baseline microbial richness, the abundance of microbial species capable of 7α/β-dehydroxylation of primary bile acids to secondary bile acids correlated with preferential response of IBD patients to anti-TNF therapy^[139]^. In the same study^[139]^, the identification of two metacommunities amongst the faecal microbiota of IBD patients revealed that those showing dominance of Firmicutes species including *F. prausnitzii* and *Ruminococcus bromii* had higher rates of clinical remission and endoscopic remission following the anti-cytokine therapy. Relapsing disease and poor response to treatment with TNF inhibitors was associated with disturbances to SCFA-producing bacteria including *Lachnospiraceae* and *Ruminococcaceae* which was replicated across two cohorts of CD and UC patients^[140]^. In children with CD, sustained response to infliximab treatment was associated with higher abundances of faecal concentrations of amino acids, including L-aspartic acid, as well as linoleic acid and L-lactic acid, and higher abundances of *Streptococcus*, *Staphylococcus*, *Methylobacterium* and *Sphingomonas* at baseline^[141]^. In a proof-of-concept study, Busquets *et al*. designed an algorithm to identify bacterial biomarkers of non-responding IBD patients to anti-TNF agents involving 38 IBD patients^[142]^. Based on this, patients prone to respond have high counts of *F. prausnitzii* and *Ruminococcus* and a low abundance of *Methanobrevibacter smithii*. The algorithm was reported to have a positive predictive value of 100% and a negative predictive value of 75% for predicting response to treatment. However, this would need to be tested in much larger patient cohorts. A systematic review of 10 studies involving biologic therapies reported that the gut microbiota of IBD patients who responded to anti-TNF agents (8 studies) had higher α-diversity and greater relative abundances of genera from the Clostridiales order, including *Faecalibacterium*, *Roseburia* or *Clostridium* (butyrate-producers) either at baseline or during follow-up^[143]^.

It thus appears that the SCFA-, and especially butyrate-producing capacity of the gut microbiome is an important consideration for anti-TNF treatment efficacy.

## VEDOLIZUMAB

Vedolizumab is a monoclonal antibody that blocks the binding of α_4_β_7_ integrin, expressed on a subset of gastrointestinal-homing T lymphocytes, to mucosal addressin cell adhesion molecule-1 (MAdCAM-1), expressed by endothelial cells of the GIT - this action selectively prevents the infiltration of lymphocytes into the inflamed gastrointestinal submucosa^[144,145]^. Approved in recent years, it is used to treat patients with moderate to severe IBD who have not achieved treatment success with at least one conventional therapy^[144]^. It can be intravenously or subcutaneously administered. A systematic review of vedolizumab efficacy for UC involving 9 studies concluded that most studies reported clinical response and clinical remission in UC patients^[146]^.

### Gut microbiome biomarkers of responders and non-responders to vedolizumab

Ananthakrishnan *et al*. demonstrated the ability to predict treatment response to anti-integrin therapy using the gut microbiome and clinical data of IBD patients^[147]^. Their study highlighted the role of both microbial taxonomy and functional pathways that may be relevant to treatment. Specifically, in CD patients who achieved remission, gut microbiota alpha diversity was significantly higher at baseline compared with those who failed to achieve remission. For UC patients, the difference in alpha diversity between responders and non-responders at baseline was not significant, but a wider range of baseline community diversity was noted for those who achieved remission. Furthermore, for CD patients, beta diversity within the responders’ group at baseline was lower than that within the non-responders group. The relative abundance of the species *Roseburia inulinivorans* and a *Burkholderiales* species in CD patients was significantly greater at baseline in those who responded to therapy. *R. inulinivorans* is a butyrate producer^[148]^. In CD patient responders, 13 microbial pathways were also enriched at baseline, including branched-chain amino acid biosynthesis. In terms of the longitudinal trajectory of the microbiome, the following five species decreased in relative abundance in those who achieved remission: *Bifidobacterium longum*, *Eggerthella*, *R. gnavus*, *R. inulinivorans*, and *Veillonella parvula*. However, in those who did not achieve remission, *Streptococcus salivarium* significantly increased in relative abundance. By follow-up, several microbial pathways were significantly decreased in CD patients who had achieved remission. These included many tricarboxylic acid pathways and a nicotinamide adenine dinucleotide salvage pathway, which the authors suggest indicates decreased oxidative stress in responders. Also decreased was O-antigen building blocks biosynthesis in *E. coli*, though *E. coli* abundance did not change. The changes were not as marked in UC patients, where those who achieved remission showed higher relative abundances of three pathways by follow-up: polyamine biosynthesis, non-oxidative pentose phosphate pathway, and sucrose degradation compared with non-responders. To determine if strain variability at baseline impacts treatment outcome, the authors employed a strain-resolution approach focusing on the pathways that differentiated responders from non-responders and found that CD patient responders harboured a cluster of unique single nucleotide polymorphisms (SNPs) in L-arginine biosynthesis pathways predominantly contributed by *B. longum* and *Dialister invisus*. In UC baseline samples, responders had a more diversified group-specific SNP profile found in the uridine monophosphate biosynthesis pathway and pentose phosphate pathway and the differentiating species contributing to the SNPs included *B. longum*, *Ruminococcus torques* and *E. coli.* Using baseline clinical data, microbial taxa, particularly species-level data, and pathways in a neural network algorithm enabled the prediction of treatment response, achieving a true positive discovery rate of > 80% with a less than 25% false negative discovery rate. Interestingly, other factors that compounded treatment response success were shorter duration of the disease, a diagnosis of CD as opposed to UC, and non-exposure to anti-TNF therapies in the past.

Lee *et al*. reported that in IBD patients receiving vedolizumab therapy, increased relative abundances of three species, *B. ovatus*, *Bacteroides stercoris*, and *B. longum*, in the baseline microbiota, were linked to early clinical remission^[139]^. Interestingly, in a mouse model of IBD, *B. ovatus* monotherapy was more effective and consistent for ameliorating colitis than faecal microbiota transplantation (FMT)^[149]^.

### Ustekinumab

Ustekinumab is a monoclonal antibody that is active against the shared p40 subunit of the proinflammatory interleukins IL-12 and IL-23, whose signalling pathways play an important role in IBD pathogenesis^[150]^. Approved for CD treatment in 2016, it is administered subcutaneously or intravenously. It has proven to be effective and safe for CD patients who are intolerant to anti-TNF therapy or when anti-TNF therapy has failed^[151]^.

### Gut microbiome biomarkers of responders and non-responders to ustekinumab

Doherty *et al*. investigated if the faecal microbiota could be used as a biomarker for response to ustekinumab therapy in patients with CD^[152]^. Researchers found that the relative abundance of *Escherichia/ Shigella* spp. was lower at baseline in subjects who achieved remission after 6 weeks of treatment (270-90 mg ustekinumab subcutaneously) compared to subjects with active CD. In addition, *Faecalibacterium* and *Bacteroides* were significantly more abundant in the baseline stool samples from the subjects who were in remission six weeks after treatment than patients with active CD. The beta-diversity of the microbiotas was significantly different between responders and non-responders before treatment. Additionally, the median baseline microbial diversity in subjects with remission after 6 weeks was 1.7 times higher than in treated subjects with active CD (*P* = 0.020). *Faecalibacterium* frequently occurred in their random forest models and was associated with health, comprising up to 5% of the relative abundance in healthy individuals, and was generally rare in CD patients. Each subject in remission 6 weeks after ustekinumab therapy had measurable *Faecalibacterium* present in their baseline samples. Interestingly, a lower dose of ustekinumab (45 mg) to treat psoriasis had minimal impact on the gut microbiome^[153]^, indicating that the impact of ustekinumab on the microbiome may be dose-dependent.

## NUTRITION

### Exclusive enteral nutrition

EEN describes the use of a nutritionally replete liquid diet that is exclusively fed to CD patients for a period of generally 8 weeks^[154]^, after which foods are slowly introduced^[155]^. European guidelines recommend EEN as a first-line therapy for pediatric patients with luminal CD^[156]^. EEN formulas can differ in their protein and fat content and are classified as elemental, semi-elemental or polymeric, where elemental formulas are generally low in fat, and contain amino acids and glucose polymers; semi-elemental formulas contain primarily medium chain triglycerides as fat, peptides of varying chain lengths, simple sugars, glucose polymers, or starch; and polymeric formulas contain predominantly long-chain triglycerides, complex carbohydrates and intact proteins^[157]^. EEN is reported to induce remission in up to 80% of patients^[158]^. It is postulated that the efficacy of EEN is due to multiple mechanisms, from altering gut microbiota composition and metabolism to impacting immunity, minimising xenobiotic exposure, and improving nutritional status, as examples^[159]^.

### Impact of EEN on the gut microbiota

EEN has been shown to modulate gut microbiome structure, changing the relative abundances of many bacterial families, including *Clostridiaceae*, *Eubacteriaceae*, and *Bacteroidaceae*^[160]^. Many studies have also reported that EEN reduces gut microbial diversity, which has been the topic of review and systematic review^[157,158]^ and is attributable to the reduced components of EEN compared to a regular diet^[161]^. Indeed, Quince *et al*. reported that for every 10 days on EEN, 0.6 genus diversity equivalents were lost^[162]^. Diederen *et al*. also reported that EEN reduced gut microbiota diversity in 43 children with CD and decreased amino acids, trimethylamine, and cadaverine toward control levels^[161]^. Furthermore, the reduced microbial metabolism of bile acids observed in the CD pediatric patients was partially normalised during treatment. In contrast, Lv *et al*. reported that species-level diversity in pediatric CD patients increased toward levels seen in controls^[163]^, while Jones *et al*. reported no change in diversity following treatment^[164]^. But Lv *et al*. did observe that EEN improved the imbalance of primary and secondary bile acid metabolism in CD pediatric patients such that secondary unconjugated bile acids increased and resembled levels in healthy controls following EEN^[163]^. It has been proposed that EEN-induced remission in patients could be partially due to microbial bile acid modifications^[165]^. Following EEN treatment, the enriched bile acids hyocholic acid and α-muricholic acid were strongly associated with decreased CD symptom severity^[165]^. These bile acids significantly correlated with increased relative abundances of *Clostridium innocuum* and *Hungatella hatherwayi*, which express the enzymes 3β-hydroxysteroid dehydrogenase and 5β-reductase. In the same study, hyocholic acid suppressed TNF-α production by CD4+ T cells in peripheral blood mononuclear cells from CD patients, while intraperitoneal injection of hyocholic acid attenuated DSS-induced colitis in mice.

EEN has also been reported to significantly increase Firmicutes while reducing Proteobacteria in children^[163]^ and adults^[166]^ with CD. In the latter study^[166]^, EEN was also associated with significantly increased relative abundances of five species, including *Ruminococcus*, *Lachnospiraceae*, *Anaerotruncus*, *Flavonifracter*, and *Novosphingobium*, accompanied by relative changes in faecal SCFAs.

### Gut microbiome biomarkers of responders and non-responders to EEN

A few studies have examined the gut microbiota signatures that are associated with treatment response and sustained remission following EEN. In a study involving 10 pediatric CD patients, Dunn *et al*. reported that 9 achieved clinical remission by week 12 (the conclusion of EEN therapy), but 4 of these patients experienced clinical relapse by week 24^[167]^. Interestingly, in these 4 relapsing patients, alpha diversity at baseline was lowest. In those who sustained remission, EEN decreased diversity by week 12 of treatment, a response that was not observed in those who failed to achieve remission; in fact, in this group, EEN increased diversity by week 12. At baseline, Proteobacteria was higher in those who did not sustain a response to EEN and increased in prevalence over the course of treatment. Those who sustained remission also had a number of *Akkermansia muciniphila* strains and *Bacteroides*, such that the authors propose that patients with a high prevalence of one or more strains of *A. muciniphila* at the onset of treatment would be more likely to benefit from EEN treatment due to early improvements in intestinal epithelial function. Based on the results, a Bayesian model trained to differentiate baseline samples into sustained response and non-sustained response achieved 80% classification accuracy. The predictive success of the model is due to the “occurrence patterns” of specific predominant OTUs in baseline samples, namely *A. muciniphila*, *Bacteroides* (including *B. fragilis* and *B. ovatus*), *Lachnospiraceae*, and *Ruminococcaceae* in patients who achieve sustained remission. In contrast, the predominant strains in patients who failed to achieve sustained remission are generally members of *Bacteroides* (including *B. plebeius*), *Enterobacteriaceae* (including *Klebsiella*), and *Prevotella*. In a follow-up study, a random forest model using microbial abundances, species richness and Paris disease classification proved the most informative in predicting response to EEN^[164]^. Another study reported that *Dorea longicatena*, *Blautia obeum* and *E. coli* found in non-responders at baseline represent examples from the predictive signature of no response to EEN^[161]^.

## NON-CONVENTIONAL THERAPY

### Hematopoietic stem cell transplantation

Multipotent hematopoietic stem cells can be isolated from bone marrow, umbilical cord or peripheral blood and have self-renewal capabilities and the capacity to differentiate into immune and blood cells^[168]^. They can also migrate to damaged tissue and differentiate into immune-modulatory or epithelial cells, thus enabling them to restore mucosal tissue and integrity^[168,169]^. HSCT is thus a promising treatment in cases of severe refractory CD, where it is proposed to “establish a new immune system in the intestine of IBD patients”^[170]^. In a multicentre retrospective study evaluating the clinical use and outcomes of autologous HSCT performed in 19 European Society for Blood and Marrow Transplantation (EMBT) centres across seven countries involving 82 patients, Brierley *et al*. concluded that it was relatively safe and effective in controlling otherwise treatment-resistant CD^[171]^. Given the role of the gut microbiota in modulating host immunity, there is growing interest in the role of the gut microbiota in HSCT^[172]^. Furthermore, changes to the intestinal environment triggered by HSCT will undoubtedly alter the gut microbiome.

### Impact of HSCT on the gut microbiota and gut microbiome biomarkers of responders and non-responders to HSCT

In patients with X-linked inhibitor of apoptosis protein (XIAP) deficiency and who are suffering from refractory IBD, HSCT was reported to improve disease symptoms, significantly improve the pediatric ulcerative colitis activity index, and improve gut microbiota dysbiosis observed before treatment such that the microbiota resembled that of healthy family members following HSCT^[173]^. Microbial profiles were also reported to differ in patients with active CD pre- and post-HSCT (non-responders) and in those with inactive disease post-HSCT (responders)^[174]^. Non-responders to treatment exhibited post-treatment microbiotas enriched with members belonging to *Enterococcus*, *Fusobacterium*, *Haemophilus*, *Megasphaera*, and *Campylobacter*, while treatment responders were enriched in *Alistipes*, *Akkermansia Roseburia*, *Christensenellaceae*, *Oscillibacter* and *Odoribacter* post-treatment. Interestingly, functional modules differentially abundant in responders and non-responders post-HSCT revealed enrichment of basic biosynthesis processes in responders, while pathways involved in sulphur transport and other ion transport systems (e.g., molybdate and nickel) were enriched in non-responders post-HSCT. However, it was not possible to predict response to treatment based on pretreatment microbiota samples as there were no significant differences in bacterial community structure, richness, or diversity in baseline samples.

### Mesenchymal stem cell therapy

Mesenchymal stem cells (MSCs) are also multipotent and can differentiate into multiple mesoderm lineage cell types including osteoblasts, adipocytes, myocardiocytes, and chondrocytes^[168,175-177]^ and they are immunomodulatory, being capable of downregulating mucosal immune reactivity^[168,178]^ and can thus control inflammation^[179]^ fostering the repair of damaged mucosal tissue. They can be isolated from bone marrow, adipose tissue and umbilical cord, and unlike hematopoietic stem cells, they have low immunogenicity^[168]^. In clinical trials, MSCT has proven to be safe and effective for the treatment of refractory CD^[180-182]^ and UC^[183-185]^, although the long-term efficacy of MSCT must yet be verified^[186]^.

### Impact of MSCT on the gut microbiota

In a DSS-mouse model of colitis, researchers compared the effectiveness of MSCs derived from induced pluripotent stem cells or adipose-derived MSCs and reported that both cell types significantly reduced lesion scores and gut inflammation and partially restored the gut microbiome to resemble that of healthy mice^[187]^. He *et al*. reported a similar finding in the study of bone marrow-derived MSC for the prevention of cancer in a colitis-associated cancer mouse model^[188]^. Specifically, MSC treatment resulted in a significant reduction in the Firmicutes/Bacteroidetes ratio, increased the relative abundance of potentially beneficial bacteria including *Parabacteroides*, *Staphylococcus*, *Acetifactor*, *Intestinimonas*, and *Candidatus Saccharimonas*, and reduced potentially pathogenic bacteria including *Bilophila* and *E. branchy*. In another mouse model of colitis induced with TNBS, human umbilical cord-derived MSCs improved disease symptoms and increased survival rates^[189]^. MSCT prevented the gut microbiota dysbiosis observed in the TNBS group and increased alpha diversity, decreased Proteobacteria, and increased Bacteroidetes, Firmicutes, and Tenericutes. It also decreased sulphur metabolism compared to the TNBS group.

In a human study, 8 MSC infusions in CD patients dramatically relieved clinical symptoms^[190]^. In terms of the microbiota, the Cyanobacterium phylum was depleted, while the Nitrospirota phylum was enriched after the 8 infusions. The genus *Cetobacterium* was significantly enriched after 8 infusions and the CD activity index score correlated strongly with *Cetobacterium* abundance. Interestingly, *Cetobacterium somerae* has been reported to improve glucose homeostasis and increase insulin levels in larval zebrafish^[191]^, and fermentate of the bacterium has been shown to improve antiviral immunity as well as gut and liver health in zebrafish^[192]^.

## CONCLUSION AND FUTURE DIRECTIONS

The field of science that investigates the interplay between IBD therapeutics and the gut microbiome is still very much in its infancy. However, the studies that have been conducted to date reveal that this is an exciting and essential field of investigation that could lead to the development of future novel treatments composed of IBD therapeutics and microbial-based therapies that simultaneously focus on the immune response of the patient and the gut microbiome to improve treatment efficacy [Table 1]. The interindividual variability of the gut microbiome means that such therapies could be developed as personalised, precision-based treatments. Indeed, the screening of IBD patient stool samples at diagnosis could enable the design of better treatments. For example, strategies that increase baseline gut microbiota alpha diversity or increase the absolute abundance of particular gut commensals, e.g., butyrate producers, could serve to improve treatment responses given that such signatures are common features of responders to IBD therapies [Figure 1], leading to the design of IBD-specific probiotics. For 5-ASA azo-bonded therapeutics, increasing the abundance of azo-reducing bacteria that release the active moiety of the therapeutic in the intestine may serve to improve the effectiveness of treatment in patients with low levels of gut microbial azoreductases. Administering *A. muciniphila* before the commencement of EEN could ensure that remission is sustained following treatment. Furthermore, FMT could be used to convert non-responders to responders by performing FMT in these patients with a “responder faecal microbiota” or healthy faecal microbiota. The efficacy of such an approach has already been proven with the step-up FMT strategy, which consists of scheduled FMTs (single or multiple) followed by treatment with steroids, TNF inhibitors, or EEN^[193]^. It has proven efficacious for the treatment of UC and CD^[194-196]^. Interestingly, donor microbiota tends to be derived from healthy young people aged from 5 years to 24 years. Cui *et al*. propose that one or two FMTs prior to therapeutic administration remodel the intestinal microbiota, thus improving the patient’s ability to respond to other therapies^[193]^. Indeed, step-up FMT patients displayed increased gut microbial diversity and a composition trending toward that of donors following FMT^[194]^.

Some studies have reported the clinical benefits of using microbial therapies as adjuncts to conventional therapies, yielding promising results - though these studies did not use personalised, precision-based data. For example, *E. coli* Nissle 1917 as an add-on therapy to mesalazine in UC patients resulted in a significantly higher number of patients displaying clinical response at four weeks (*P* = 0.04), and endoscopic remission at eight weeks (*P* = 0.03)^[197]^. A consortium of live probiotics as an adjunct to 5-ASA and/or immunosuppressants in relapsing UC patients improved rectal bleeding and reinduced remission in relapsing patients after eight weeks compared to the drug treatments alone^[198]^. A meta-analysis of randomised controlled trials reported that probiotics in conjunction with 5-ASA can increase the clinical remission rate of active UC over the drug alone^[199]^. We have already seen that *L. plantarum* with tacrolimus is capable of improving gut microbiome diversity and promoting the therapeutic effect of the drug in a mouse model of colitis^[102]^.

However, the studies to date have also highlighted potentially concerning interactions between the gut microbiome and IBD therapeutics. For example, the bacterial enzyme steroid-17,20-desmolase can convert the IBD therapeutic prednisone to a metabolite that causes the proliferation of prostate cancer cells^[86]^. Studies of this nature highlight the importance of investigating the metabolomics of gut microbiota-IBD therapeutic interactions and that the activity of the gut microbiome on IBD therapeutics should be considered in the safety and adverse effects of these drugs. This is a field that requires much deeper investigation. Furthermore, the impact of these therapeutics on the gut resistome is also an important consideration, especially in cases where they have antimicrobial effects. Methotrexate has been shown to select for antibiotic resistance genes^[70]^. Antibiotic resistance is a major global health crisis, with antibiotic stewardship programs now mandating the safe and appropriate use of antibiotics; however, IBD therapeutics, although designed to alter immune function, should be assessed for their impacts on the gut resistome.

Gut microbial metabolism can also reduce therapeutic efficacy by degrading the drugs to less potent metabolites, as is in the case of the calcineurin inhibitor, tacrolimus^[100]^. Furthermore, the gut microbiome may be capable of cleaving TNF inhibitors^[125]^. Thus, further studies are warranted to determine the impact of the microbiome on the stabilities of IBD therapeutics, which may lead to the design of adjunct molecules that inhibit microbial degradation.

While stem cell therapy is emerging as a viable option for refractive IBD, more studies are warranted to investigate the impact of these treatments on the gut microbiome and functionality and also to determine how microbiome composition and structure can impact stem cell therapy efficacy.

Many studies to date have investigated the interplay between single IBD therapeutics and the gut microbiome; however, IBD patients may receive multiple treatments during active disease, and this is another consideration that should be taken into account by investigating the impact of combinations of IBD therapeutics on the gut microbiome and vice versa^[200]^. Indeed, Tourret *et al*. reported that a combination of immunosuppressive therapeutics increased colonisation of a uropathogenic strain of *E. coli* in mice^[88]^.

In conclusion, the studies discussed in this review demonstrate the importance and value of continued research and the use of integrated studies for investigating the interplay between IBD therapeutics and the gut microbiome. Technologies such as metabolomics that enable investigation into the xenobiotic-metabolizing capabilities of gut microbes are still in their infancy but will be invaluable in the future for understanding the role the microbiota plays in the metabolism of IBD therapeutics and how these therapeutics impact microbiota metabolism. Utilising gut microbiome data in conjunction with IBD therapies will ultimately result in more effective, personalized, precision treatments that achieve faster remission and increase patients’ quality of life.

## DECLARATIONS

### Copyright

© The Author(s) 2023.
